# Supplementary figures and images for: A retrospective study on beta-blocker use and outcomes in hematopoietic stem cell transplant patients
Source: PeerJ. 2025 Aug 8;13:e19822. doi: 10.7717/peerj.19822 (PMC12338055; doi:10.7717/peerj.19822)

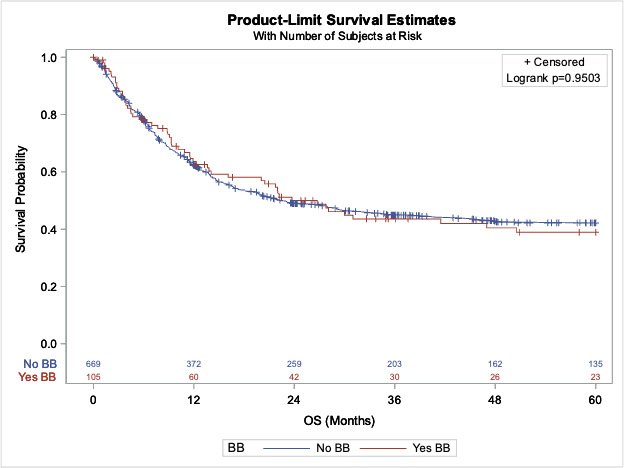

Supplement: Supplemental Information 1 — Kaplan–Meier curves of (A) overall survival, (B) non-relapse mortality (NRM)-related rate, (C) acute GvHD-free rate, (D) chronic GvHD-free rate, and (E) relapse-free rate for treatment groups defined based on whether patients had at least four consecutive days of BB use both before and after transplantation (peri-HCT): patients who did not receive a beta blocker (No BB; blue) and patients who received a beta blocker (Yes BB; red). Overall group differences were evaluated using log-rank tests. [file peerj-13-19822-s001.png]

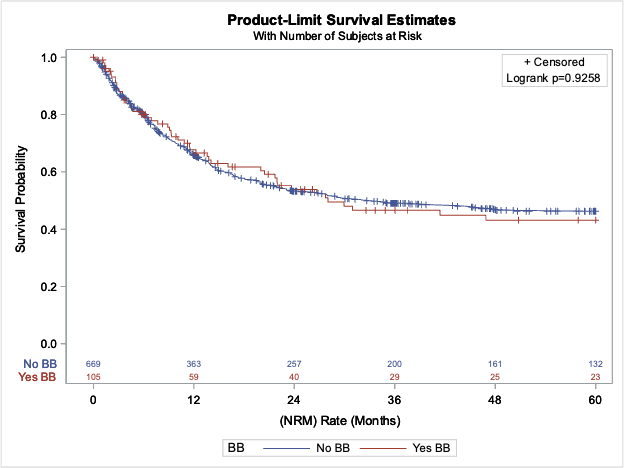

Supplement: Supplemental Information 2 — Kaplan–Meier curves of (A) overall survival, (B) non-relapse mortality (NRM)-related rate, (C) acute GvHD-free rate, (D) chronic GvHD-free rate, and (E) relapse-free rate for treatment groups defined based on whether patients had at least four consecutive days of BB use both before and after transplantation (peri-HCT): patients who did not receive a beta blocker (No BB; blue) and patients who received a beta blocker (Yes BB; red). Overall group differences were evaluated using log-rank tests. [file peerj-13-19822-s002.png]

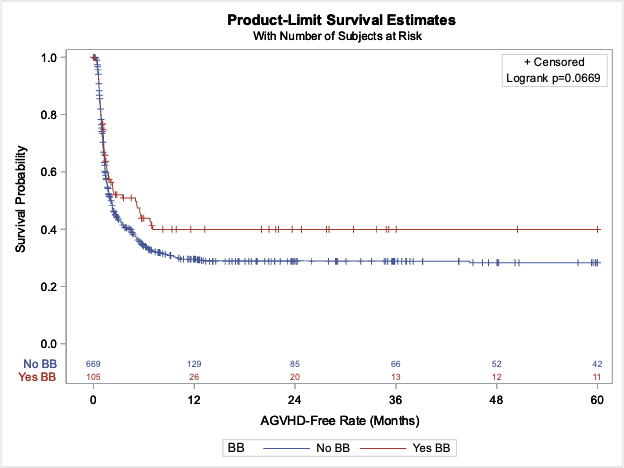

Supplement: Supplemental Information 3 — Kaplan–Meier curves of (A) overall survival, (B) non-relapse mortality (NRM)-related rate, (C) acute GvHD-free rate, (D) chronic GvHD-free rate, and (E) relapse-free rate for treatment groups defined based on whether patients had at least four consecutive days of BB use both before and after transplantation (peri-HCT): patients who did not receive a beta blocker (No BB; blue) and patients who received a beta blocker (Yes BB; red). Overall group differences were evaluated using log-rank tests. [file peerj-13-19822-s003.png]

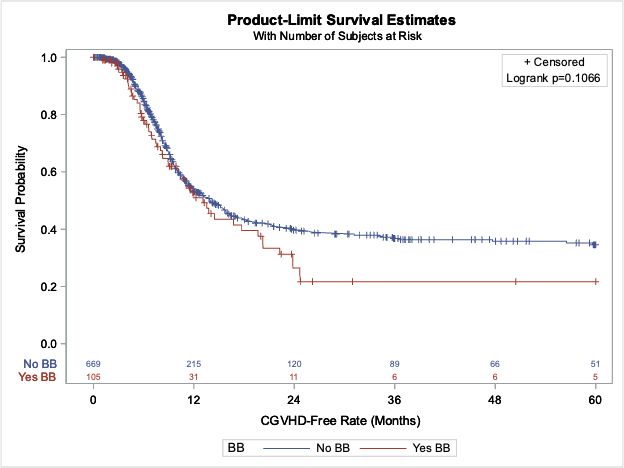

Supplement: Supplemental Information 4 — Kaplan–Meier curves of (A) overall survival, (B) non-relapse mortality (NRM)-related rate, (C) acute GvHD-free rate, (D) chronic GvHD-free rate, and (E) relapse-free rate for treatment groups defined based on whether patients had at least four consecutive days of BB use both before and after transplantation (peri-HCT): patients who did not receive a beta blocker (No BB; blue) and patients who received a beta blocker (Yes BB; red). Overall group differences were evaluated using log-rank tests. [file peerj-13-19822-s004.png]

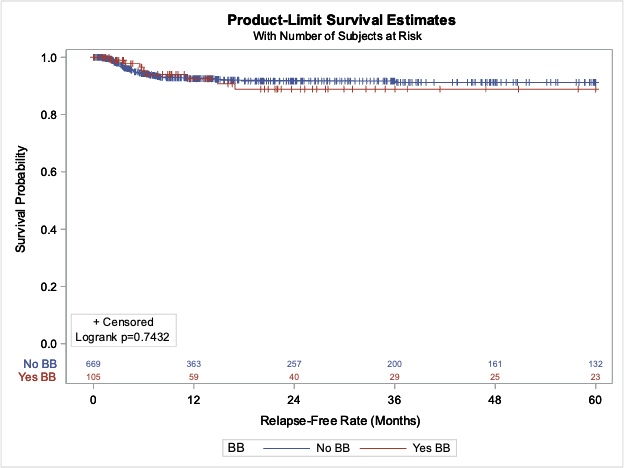

Supplement: Supplemental Information 5 — Kaplan–Meier curves of (A) overall survival, (B) non-relapse mortality (NRM)-related rate, (C) acute GvHD-free rate, (D) chronic GvHD-free rate, and (E) relapse-free rate for treatment groups defined based on whether patients had at least four consecutive days of BB use both before and after transplantation (peri-HCT): patients who did not receive a beta blocker (No BB; blue) and patients who received a beta blocker (Yes BB; red). Overall group differences were evaluated using log-rank tests. [file peerj-13-19822-s005.png]
